# Bidirectional Interaction Between Cancer Cells and Platelets Provides Potential Strategies for Cancer Therapies

**DOI:** 10.3389/fonc.2021.764119

**Published:** 2021-10-14

**Authors:** Liuting Yu, Yao Guo, Zhiguang Chang, Dengyang Zhang, Shiqiang Zhang, Hanzhong Pei, Jun Pang, Zhizhuang Joe Zhao, Yun Chen

**Affiliations:** ^1^ Edmond H. Fischer Translational Medical Research Laboratory, Scientific Research Center, The Seventh Affiliated Hospital, Sun Yat-Sen University, Shenzhen, China; ^2^ Department of Urology, The Seventh Affiliated Hospital, Sun Yat-sen University, Shenzhen, China; ^3^ Department of Pathology, University of Oklahoma Health Sciences Center, Oklahoma City, OK, United States

**Keywords:** platelets, thrombosis, cancer cells, cancer-platelet crosstalk, cancer treatment

## Abstract

Platelets are essential components in the tumor microenvironment. For decades, clinical data have demonstrated that cancer patients have a high risk of thrombosis that is associated with adverse prognosis and decreased survival, indicating the involvement of platelets in cancer progression. Increasing evidence confirms that cancer cells are able to induce production and activation of platelets. Once activated, platelets serve as allies of cancer cells in tumor growth and metastasis. They can protect circulating tumor cells (CTCs) against the immune system and detachment-induced apoptosis while facilitating angiogenesis and tumor cell adhesion and invasion. Therefore, antiplatelet agents and platelet-based therapies should be developed for cancer treatment. Here, we discuss the mechanisms underlying the bidirectional cancer-platelet crosstalk and platelet-based therapeutic approaches.

## 1 Introduction

Platelets are small anucleate blood cells (2–4 µm) released from bone marrow megakaryocytes with a normal number ranging from 150×10^9^/L to 350×10^9^/L in the bloodstream. They not only play a crucial role in hemostasis and thrombosis formation but also modulate inflammatory response through interacting with granulocytes and pathogens (1). It is generally accepted that tumors behave like chronic or non-healing wounds and trigger inflammation (2, 3). As the first responder during chronic inflammation and cancer progression, platelets have such advantages as small size, the large numbers in the bloodstream and versatile biophysical properties including adhesion, aggregation, and streamline migration (4). Activated platelets can change their shape and release α granules, dense granules or lysosomal granules in response to different stimuli. These granules contain various cytokines or molecules with distinct functions (5).

Ever since Armand Trousseau described the relationship between cancer and abnormal blood coagulation in 1865, numerous studies have showed that platelets contribute to cancer-associated thrombosis and influence the outcomes of cancer treatment. Cancer cells can activate platelets and cause their aggregation in the circulation, while platelets help to maintain the integrity of tumor vasculature and participate in multiple steps of metastasis (6). Hence, platelets are excellent biomarkers for liquid biopsy to improve diagnostic and prognostic accuracies (6). Furthermore, antiplatelet agents have a great potential in anti-cancer therapies (7, 8).

This review addresses the bidirectional interaction between platelets and cancer by highlighting facts that elevated platelet counts in patients with malignancy predict adverse prognosis and short survival and that cancer cells induce platelet production, activate platelets and alter their functions. Potential strategies for platelet-based cancer therapies are also discussed.

## 2 Correlation Between Increased Platelet Counts and Cancer Prognosis

From clinical data, many cancer patients were reported to have high platelet counts. Generally, thrombocytosis is defined as more than 400×10^9^/L of platelet counts. The frequencies of pretreatment thrombocytosis varied according to cancer types, 4.0% to 21% in gastric cancer patients (9, 10), 9.8% to 13.2% in colorectal cancer patients (11, 12), and 3.7% to 18.2% in breast cancer patients (13, 14). Increased platelet counts usually indicate worse prognosis and shorter survival in patients with malignant diseases (15–18). For example, Zhou et al. investigated 6754 ovarian cancer patients and found that elevated pretreatment platelet counts denoted poor survival outcome and unfavorable clinicopathological parameters (16). In gastric cancer, patients with thrombocytosis had worse overall survival (HR 1.57, 95% CI 1.36–1.81) and higher likelihood of recurrence (OR, 2.28; 95% CI, 1.55–3.35) (19). Although only 2.4% patients with oesophageal adenocarcinoma had paraneoplastic thrombocytosis, such patients had a higher rate of mortality (86%) and lymph node metastasis (69%) than patients with normal platelet counts (50% and 31%, respectively). The former patients died with a median survival time of 23.2 months while the latter died with a median survival time of 76.9 months (20). These data demonstrate that platelets are likely to take an important part in progression. Interestingly, a new finding denoted that upper tract urothelial carcinoma patients with both high platelet counts and programmed cell death ligand-1 (PD-L1) positivity had shorter metastasis-free survival and overall survival, demonstrating PD-L1 expression might synergize with platelet count in modulating cancer development (21).

Thrombocytosis is significantly associated with cancer metastasis. For ovarian patients with thrombosis before surgery, anticoagulant drugs was used to inhibit the thrombosis formation and cancer metastasis (22). Latest data from patients who had undergone radical hysterectomy and pelvic lymphadenectomy showed that thrombocytosis could be one predictor of pelvic lymphatic metastasis in the early squamous cervical cancer (23). In addition, platelet counts correlated with tumor invasion and distant metastasis in gastric cancer (24), colorectal cancer (17, 25) and pulmonary malignancy (26). Thus, platelets seem to actively participate in cancer dissemination, which may be a main reason for adverse prognosis in cancer patients with thrombocytosis.

Different researchers set the cutoff values of thrombocytosis varying from 270 to 450×10^9^/L in their studies. This discordance may lead to between-study heterogeneity and affect the significance of results (16). For example, Shimada (27) and Ling (28) defined thrombocytosis as platelet count over 293×10^9^/L in esophagus tumor. They found thrombocytosis appeared in approximately 20% patients, whereas Aminian (29) and Dutta (30) reported a 3.4–4.46% incidence based on the 400×10^9^/L cutoff. In fact, it may be useful to adopt specific cut-off values according to the features of tumors. For instance, lower cutoff for platelet counts (300×10^9^/L) was more informative to predict prognostics of inflammatory breast cancer (31). Moreover, a cohort study demonstrated that the risk of cancer in men with a platelet count over 325×10^9^/L exceeded 3% while the risk in women with a platelet count over 375×10^9^/L exceeded 2.8%. This finding could be a cue for earlier diagnosis of cancer in patients with platelet counts above these values (32). Since platelet counts were also affected by age and sex (33, 34), age/sex-specific ranges of platelet counts were introduced to better predict the risk of total mortality (35). More data will help to define a clear relationship between the platelet counts and the cancer incidence. Furthermore, using platelet counts as indicators for diagnosis and prognosis should take account of patient conditions.

Altogether, elevated platelet counts can be recognized as a risk marker in certain types of cancers (16, 17, 36). However, it was found that platelet count was not statistically significantly associated with colorectal cancer patient survival though higher platelet counts were observed in higher tumor stage (37). Ishibashi et al. also suggested that platelet count was non-independent prognostic factors for overall survival in esophageal squamous cell carcinoma (38). Studies showed that a combination of platelet counts and other factors often has higher prediction value than a single index, such as platelet-to-lymphocyte ratio (PLR) (39–41) and hemoglobin/albumin/lymphocyte/platelet (HALP) levels (42). Aside from neutrophil–lymphocyte ratio (NLR) and lymphocyte–monocyte ratio (LMR), patients with high PLR were at higher risk of distant metastases and worse prognoses in renal cell cancer (43), cervical cancer (44), bladder cancer (45), colorectal adenocarcinoma (46), head and neck squamous cell carcinoma (47), and gastric cancer (48). Recently, a novel parameter neutrophil/platelet/lymphocyte/differentiation score (NPLDS) has been introduced to accurately predict the prognosis of chemotherapeutic response in advanced gastric cancer (49). Collectively, the detection of PLR, HALP, and NPLDS is valuable in the evaluation of cancer patient outcomes while the underlining mechanisms need further investigation.

## 3 Impacts of Cancer on Platelets

Clinical data demonstrated that cancer patients had a higher risk of venous thrombosis than the healthy individuals do (50). Thrombosis has been reported to be the second leading cause of malignancy-associated death (51). Recently, the relationship between thrombosis and cancer reviewed by Plantureux et al. indicated that cancer cell-induced platelet production, activation and function alteration might be the major reasons of thrombosis (5). Zhang et al. have found that activated platelets from patients with colorectal cancer could stimulate the formation of neutrophil extracellular traps (NETs) and ultimately enhance procoagulant activity (52). Cancer cell-platelet interaction incited platelet-derived extracellular vesicles (EVs) release and fibrin formation, thus inducing thrombus formation under shear flow (53).

### 3.1 Platelet Production

Cancer cells can induce platelet production. Early studies showed that overexpression of interleukin-1β (IL-1β) (54) and interleukin-6 (IL-6) in malignant diseases (55, 56) was related to thrombocytosis. In subsequent studies, thrombocytosis was found to be caused by IL-6 in Granulocyte-Colony-Stimulating Factor (G-CSF)-producing tumors and by both granulocyte-CSF and IL-6 in Granulocyte Macrophage-Colony-Stimulating Factor (GM-CSF)-producing tumors (57). Studies have shown that tumors are able to produce thrombopoietin (TPO) (58) and IL-6 (59). TPO is the primary regulator of megakaryocyte progenitor differentiation and platelet production (60), while IL-6-induced thrombopoiesis was dependent on TPO (61). In tumor-bearing mice, treatment with IL-6 antibody abrogated thrombocytosis and augmented the therapeutic efficacy of paclitaxel (59). Knock out of IL-6 decreased the platelet counts and reduced tumor burden in a colitis-associated cancer model (62). It was noteworthy that the demand for platelets modulated murine TPO mRNA levels at least in part (63). Recently, Hill et al. suggested that tumor-derived soluble factors potentially deregulated autophagy in hematopoietic progenitors and megakaryocytes and subsequently promoted megakaryopoiesis and thrombopoiesis (64). Nonetheless, the precise molecular pathway of cancer cell-induced platelet production is yet to be defined.

### 3.2 Platelet Transcriptome and Proteome Alteration

Studies indicated that platelets from cancer patients altered their growth factor contents (65–67), RNA profile (68–71) and other parameters including platelet counts, volumes, and protein contents (72) in early stage.

#### 3.2.1 Transcriptome Alteration

It was reported that 197 platelet-related genes were significantly down-regulated in metastatic lung cancer, implying that functions of platelets may alter during cancer metastasis (70). Later, Best et al. found that mRNA sequencing of tumor-educated platelets (TEPs) was capable of distinguishing cancer patients from healthy individuals with 96% accuracy and providing the location information for six major tumors (non-small cell lung cancer, glioblastoma, colorectal cancer, pancreatic cancer, breast cancer and hepatobiliary cancer) with 71% accuracy. Moreover, TEP mRNA profiles could identify MET or *HER2*-positive and mutant *KRAS*, *EGFR* or *PIK3CA* tumors as well (68). Zhang et al. revealed that in non-small cell lung cancer (NSCLC) patients, expression levels of over 2000 platelet mRNAs and ncRNAs were changed. Some up-regulated genes including P*PBP, OST4, PF4, GP1BB* and *CCL5* were related to tumor progression. Worthy of note, histological types and tumor stages could influence the gene expression (73). Analogously, integrated bioinformatical analysis also identified twenty differentially expressed TEP mRNAs in NSCLC patients, which were associated with transport process, localization and catalyticactivity (69). Moreover, TEP mRNAs were found be associated with chemotherapeutic effects (74). Together, RNA transcriptome mapping established TEPs as promising biomarker source in liquid biopsies (75–77). Recently, Best et al. have provided a protocols to combine platelet RNA sequencing and swarm intelligence–enhanced classification algorithm development for disease diagnostics (78).

It is not quite clear how tumor cells changed TEP RNAs. One scenario is that blood platelets may take up tumor-derived microvesicles which contain numerous RNA and proteins. These microvesicles are able to promote tumor growth, angiogenesis and immune evasion (79–81). This hypothesis is supported by the fact that Glioma RNA marker EGFRvIII and prostate cancer RNA marker PCA3 were detected in patient platelets (82). It will be interesting to know how tumor-derived RNAs change platelet functions and foster tumor progression.

#### 3.2.2 Proteome Alteration

Platelets contain a wide range of proteins including chemokines, cytokines, proteases and growth factors, which are synthesized by megakaryocytes or taken up from the blood by megakaryocytes and circulating platelets (65, 72, 83, 84). Protein content in platelets was notably influenced in the presence of cancer. For example, concentrations of vascular endothelial growth factor (VEGF), platelet-derived growth factor (PDGF), platelet factor 4 (PF4) (66), connective tissue-activating peptide III (CTAPIII) and thrombospondin-1 (TSP-1) in platelets were altered depending on the types of cancer (67, 72, 85, 86). Sabrkhany et al. analyzed the platelet proteome of patients with early-stage lung and pancreas cancers. It turned out that 85 of 4384 unique proteins in platelets significantly changed their expression in cancer patients. Interestingly, 81 of these 85 proteins restored their normal level after tumor resection. Most of the over-expressed proteins were involved in inflammation, immune response, cytoskeleton organization and transport while most down-regulated proteins were linked to antigen presentation/processing and protein proteolysis. On the whole, platelet proteome was remarkably altered in cancer patients with malignant disease or early-stage and localized disease, showing that the proteome could serve as a potential cancer biomarker (87, 88). By far, a group of platelet protein biomarkers have been identified for differentiating benign adnexal lesions and ovarian cancer (FIGO stages III-IV) with high sensitivity and specificity (89). Some proteins like ACTN4 (90), WDR1 (91) and TLN1 (92) were increased while other proteins (such as PHB and SRPB6) were decreased in ovarian cancer (89). However, how tumor cells modify platelet proteome still remains unclear so far. It is possible that the megakaryocytes (93) and circulating platelets (65) both absorb proteins originated from tumor cells and therefore increase certain protein content. Some platelet proteins like ATP6Ap1 are down-regulated presumably due to the autoantibodies generated in cancer patients (87, 94). In addition, megakaryocyte functions can also be influenced by cancer cells, which consequently affect platelet content (59). Taken together, the platelet transcriptome and proteome in cancer patients are both profoundly altered. These alterations may provide valuable clues for early diagnosis of cancer. Meanwhile, the role of TEP RNAs and proteins in platelet functions also warrant further studies.

### 3.3 Platelet Activation

In the bloodstream, cancer cells perturb the surrounding microenvironment and induce abnormal platelet responses through direct cell contact or by releasing various mediators. Such mediators include ADP (95, 96), thromboxane A2 (TXA_2_) (97), tissue factor (TF) (98), thrombin (99) and matrix metalloproteinases (MMPs) (100–102). Moreover, cancer cells could directly facilitate platelet secretion of dense-granules, which was required for cancer cell–induced platelet aggregation (103). Of note, inflammatory cytokines such as TNFα, IL-6, and IL-8 and platelet agonists such as thrombin and ADP in the tumor microenvironment could promote platelet autophagy and then activate platelets, leading to thrombosis and cancer metastasis (64).

The expression level of TF was raised in many types of cancers, which was strongly associated with high incidence of thrombotic events (104–106). TF on the cancer cell surface and tumor-derived microparticles could trigger extrinsic coagulation cascade and platelet activation (53, 98). Apart from the TF-dependent mechanism, breast cancer cell-secreted extracellular vesicles could foster platelet activation, aggregation and plasma coagulation in a TF-independent manner (107). Experimental data showed that platelets could in turn facilitate TF expression and coagulating function in ovarian cancer (108).

High-mobility group box1 (HMGB1) released from tumor cells is an endogenous ligand of platelet toll-like receptor 4 (TLR4). The interplay between HMGB1 and TLR4 contributed to platelet activation and tumor spreading in mice bearing with melanoma and Lewis lung carcinoma (109).

CD97, a common tumor-associated antigen predominantly expressed in hematopoietic cells (110) and several primary and metastatic tumors (110–112), was found to be able to activate platelets and foster tumor cell invasion and metastasis *via* the LPA-mediated signal pathway (113). Ward et al. demonstrated that CD97-platelet interaction promoted platelet granule secretion, disrupted endothelial cell tight junction and further facilitated transendothelial migration (113).

Interestingly, cancer cells are capable of producing immunoglobulin G (IgG) that is quite different from B lymphocyte-derived IgG (114–116) and is reported to promote tumor growth and metastasis (117, 118). Of late, Miao et al. demonstrated that cancer cell-derived IgG could bind to platelet FcγRIIa, initiate FcγRIIa-signaling pathway and mediate platelet activation and that knocking down of IgG significantly reduced CD62P expression, aggregation, and ATP release of platelets (119).

In addition, cancer cells could induce platelet aggregation and thrombus by directly binding *via* their cell surface podoplanin (PDPN) to C-type lectin receptor type 2 (CLEC-2) on the platelets (120–122). In breast cancer and melanoma, direct interaction between cancer cells and platelets induced platelet activation, modulated the VEGF release, and regulated CXCL5 and CXCL7 discharge from platelet granules (123), which were required for granulocytes recruitment and “early metastatic niches” formation (124).

Cancer cells can indirectly activate platelets in tumor microenvironment (125). Recent studies have highlighted the involvement of neutrophil extracellular traps (NETs) in cancer-associated thrombosis (126). NETs are composed of DNA, histones, and antimicrobial proteins. Tumor-derived G-CSF promoted the blood neutrophil production and NETs formation, which led to platelet activation and thrombosis (127). Pancreatic cancer cells were reported to stimulate the generation of NETs *via* soluble protein mediators and induce platelet adhesion and active status (128). Extracellular histones could accelerate procoagulant phenotype of platelets (phosphatidylserine exposure, FV expression, P-selectin translocation) and facilitate thrombin generation *via* activating platelets (129). In addition to neutrophils, monocytes/macrophages can also generate extracellular traps in response to several stimuli (130) while whether cancer cells modulate monocytes/macrophages for platelet activation still remains unclear. NET-associated histones can promote the von Willebrand factor (a glycoprotein important for platelet adhesion and aggregation) release of endothelial cells (131). Moreover, cancer cell-derived pro-inflammatory factors upregulated TF expression of endothelial cells and monocytes (125, 132, 133), which could be attributed to platelet activation and thrombosis.

Collectively, overwhelming data suggest that cancer cells are capable of initiating platelet hyperactivation directly or indirectly thereby facilitating their development and metastasis.

## 4 Platelet–Supported Cancer Progression

Tumor-educated platelets (TEPs) can serve as good allies of cancer cells in tumor growth and metastasis through various ways.

### 4.1 Tumor Growth

Platelets secreted a number of growth factors including transforming growth factor β (TGFβ) and PDGF to foster tumor growth (134). In murine models of orthotopic ovarian cancer, platelet depletion resulted in increased tumor cell apoptosis and decreased tumor weight and microvessel density (59). After coincubation with platelets, human and murine ovarian cancer cells displayed a remarkable increase in proliferation rate in a manner dependent on the interplay between platelet released TGF1β and tumor cell receptor TGFβR1 (135). Deficiency of the TGFβ1 or TGFβR1 reduced half of the tumor size of orthotopic ovarian cancer (136). Additionally, P-selectin on activated platelets mediated platelet accumulation within solid tumors such as insulinoma and malignant melanoma, and consequently, aggregated platelets released VEGF and other growth factors to accelerate tumor growth and angiogenesis (137). *In vivo*, VEGF was found to stimulate breast cancer cell proliferation through cooperation between VEGFR-2 and integrin signaling (138). These results have confirmed the positive impacts of platelet-produced growth factors in tumor growth.

In addition to growth factors, platelet factor 4 (PF4) was found to regulate tumor microenvironment and expedite lung cancer growth (139). In addition, CLEC-2-podoplanin interaction could also modulate the proliferation of lung squamous cell carcinoma (140). Moreover, interaction between ADP and its receptor P2Y_12_ on platelets played a significant role in ovarian cancer cell proliferation. Use of P2Y_12_ antagonist could suppress primary tumor growth in the presence of platelets (141, 142). Taken together, highly activated platelets can greatly promote tumor growth *via* multiple pathways.

### 4.2 Cancer Metastasis

Studies have showed that about 90% of human cancer-related death is due to cancer metastasis (143). Cancer metastasis consists of an invasion-metastasis cascade, namely, tumor cells firstly exit their primary growth sites, survive in the circulation, extravasate at distant organ site, and lastly proliferate in the foreign microenvironments (144). Tumor cell-educated platelet (TEPs) participated in multiple steps of metastasis, helping the “villain” to do evil (134, 145). Blocking platelet activation (146) or in the absence of platelets (147), cancer metastasis was markedly repressed.

#### 4.2.1 Invasion and EMT

Platelet surface molecules (e.g. P-selectin, GPIbα, αIIbβ_3_) and secreted factors from α-granules (e.g. TGFβ, LPA, MMPs) and dense granules (e.g. serotonin, ADP, histamine) all support cancer dissemination (148). It was reported that platelet-derived exosomes and exosomal HMGB1 appeared to facilitate cancer malignancy (149). Recently, Vismara et al. showed that platelet-derived extracellular vesicles could be internalized by breast cancer cell line MDA-MB-231 and strongly potentiated cell migration and invasiveness which was associated with p38MAPK and myosin light chain (150). Intriguingly, prostate cancer stem cells (PCSCs) preferentially induced platelet aggregation, which could be attributed to increased prothrombin expression. In turn, activated platelets released stromal derived growth factor-1α (SDF-1α) to preferentially enhance PCSC invasion (151, 152).

Epithelial–mesenchymal transition (EMT) process helps cancer cells to acquire malignant cell traits including cell motility, invasiveness, and resistance to apoptosis (153). Platelets had the ability to accelerate EMT through the TGFβ signal pathway (154, 155). Labelle et al. showed that platelet-derived TGFβ and direct platelet-tumor cell contact synergized to activate TGFβ/Smad and NF-κB pathways in cancer cells, consequently enhancing lung metastasis (156). A latest report denoted that tumor necrosis factor receptor-associated factor (TRAF) family member-associated NF-κB activator (TANK)-binding kinase 1 (TBK1) acted as a mediator of platelet-induced NF-κB activation and EMT in mammary carcinoma cells (157). Podoplanin on tumor cells could mediate platelet aggregation *via* binding to CLEC2 on platelets (158) and induce TGFβ release from platelets, facilitating EMT and extravasation of tumor cells (159). Knockdown of podoplanin suppressed tumor growth and metastasis of lung squamous cell carcinoma (140). In addition to Podoplanin-CLEC2 interaction, integrin α2β1 contacting could induce TGF-β1/pSmad3 pathways as well (160). These findings confirm the importance of platelet-derived TGFβ in tumor cell aggressiveness.

Other components of platelets are also involved in the EMT process. For instance, platelet TSP1 and clusterin were able to mediate cancer cell invasiveness by regulating MMP-9 *via* the p38MAPK pathway (161). Additionally, through the cooperation with platelets, tumor cell integrin αvβ3 had the capacity to promote tumor cell extravasation and colonization in a second organ (162). Recently, researchers discovered that chemokine CCL5 and epidermal growth factor (EGF) released by platelets could increase the IL-8 secretion of tumor cells *via* initiating Akt signaling (163), while platelet-secreted CCL3 engaged its receptor CCR5 on tumor cells to upregulate MMP-1 possibly *via* the NF-κB pathway. Subsequently, tumor cells elevated their invasive and migratory abilities (164).

Lysophosphatidic acid (LPA), a crucial mediator in the tumor environment, could inhibit immune response (165) and promote cancer cell invasion and metastasis (166). Platelets are the highest producer of LPA. When platelets were activated by cancer cells, Autotaxin (ATX) with lysophospholipase D activity was released from α-granules and catalyzed the LPA generation (167). Platelet-derived LPA stimulated the secretion of IL-6 and IL-8 (168) and enhanced osteolytic bone metastasis in breast cancer (169). Further evidence indicated that ATX/LPA-signaling axis not only facilitated tumor cell motility, survival, and proliferation (170), but also induced chemoresistance by stabilizing nuclear factor-like 2 (Nrf-2) and upregulating those genes involved in drug resistance and oxidative stress response (171). These results show that the ATX/LPA-signaling axis may be highly active during tumor progression. One may postulate that blocking this signaling axis may be therapeutically important.

#### 4.2.2 Adhesion

A great number of adhesive molecules are expressed on the platelet membrane.These include integrins (e.g. aIIbβ3, α6β1, αvβ3), P-selectin, glycoprotein (GP) Ib-IX-V, and the immunoglobulin superfamily (e.g. GPVI, FcγRIIa, PECAM-1) (134, 172, 173). These molecules make platelets adhere to CTCs as well as endothelial cells and facilitate intravasation and extravasation of CTCs (134, 174). Recently, Schlesinger gave a comprehensive illustration about the role of platelet receptors in tumor cell-platelet interaction (175). For example, on platelet activation, P-selectin was translocated to the platelet surface, which contributed to platelets’ binding to endothelial cells, leukocytes and cancer cells (176). Then P-selectin mediated cancer cell metastasis (177), tumor growth and angiogenesis (137, 178). Platelet integrin α6β1 directly interacted with tumor cell ADAM9, initiating platelet activation, granule secretion and mediating tumor cell dissemination (179). Glycoprotein (GP)-VI on platelets, a key receptor for collagen, supported platelet adhesion and cancer cell arrest in the vasculature (180). It could also bind to tumor cell–expressed galectin-3. GPVI blockade *in vivo* prevented lung metastasis of colon and breast cancer cells (181). Platelet microparticles (PMPs) delivered platelet-derived receptors like CD41 to tumor cells and increased tumor cell adhesiveness to endothelium and fibrinogen. Transendothelial migration of tumor cells was consequently enhanced (182).

In summary, platelets enhance adhesion of CTCs *via* adhesive proteins thereby promoting their dissemination.

#### 4.2.3 Angiogenesis

Active proliferation and metastasis of tumor cells required new blood vessels that supplied adequate nutrients, oxygen and growth factors (183), while platelets induced early and advanced stages of angiogenesis and stabilized the newly formed vessels in tumor microenvirenment (184, 185). Upon activation, platelets released distinct α-granules containing angiogenic regulators such as VEGF, PDGF, PF4 and endostatin (134, 186). Stimulation of receptor PAR1 on platelets led to the secretion of pro-angiogenic molecules such as VEGF, whereas PAR4 stimulation contributed to the release of anti-angiogenic molecules (187). Of interest, it was found that PAR1- and PAR4-activated platelets both enhanced endothelial progenitor cells migration and tube formation, but PAR1 was more potent than PAR4 (188). Thrombin was thought to have a central role in angiogenesis (189). Thrombin/PAR1 activation could not only contribute to the upregulation of angiogenic factors, but also increase endothelial cells barrier permeability to induce angiogenesis and tumor seeding (190). VEGF, the most potent proangiogenic molecule, was significantly discharged from platelets after thrombin or TF stimulation in early breast cancer patients (191). Anticoagulants reduced VEGF release and thus weakened angiogenic potential (192). ADP-induced platelet activation resulted in increased VEGF and minimal endostatin production, suggesting that ADP release had proangiogenic effects in the tumor microenvironment (193).

Additionally, glycoprotein (GP) VI on the platelet surface contributed to vascular integrity within tumors. An antibody against platelet GPVI could cause tumor hemorrhage and augmented the effects of chemotherapeutic agents without systemic bleeding complications *in vivo* (194). PMPs that contain abundant RNA and proteins play a significant part in angiogenesis. It was reported that PMPs promoted proliferation, migration and tube formation of human umbilical vein endothelial cells, leading to angiogenesis (195). Signaling pathways of PMP-induced sprouting were involved with PI3-kinase, Src kinase and ERK (196). In lung cancer, PMPs stimulated the generation of MMPs, VEGF, IL-8 and hepatocyte growth factor (HGF), which were common angiogenic regulators for metastasis (182). All above findings highlight the importance of platelets in supporting cancer-associated angiogenesis and hence enhancing cancer metastasis.

To defend against tumor angiogenesis in the early stage, host-expressed thrombospondin 1 (TSP1) and endostatin act as negative regulators. Human platelet-derived TSP1 was acquired from megakaryocytes and stored in α-granules (83). It was thought to be a sensitive and stable marker to monitor platelet activation *in vitro* (197). In platelets of tumor-bearing mice, TSP1 was increased and thus reduced tumor growth by angiogenesis inhibition (83). However, it is unclear whether selective release of angiogenic regulators in platelets can be controlled or not. This may be a promising strategy for repressing tumor angiogenesis *via* inhibiting VEGF release or accelerating the release of endostatin and TSP1.

#### 4.2.4 TCIPA

Platelet aggregation in response to tumor cell stimulation is known as tumor cell-induced platelet aggregation (TCIPA) (198, 199). TCIPA is able to prevent circulating tumor cells (CTCs) from high shear forces and immune surveillance. Several molecules participate in TCIPA formation, including ADP, TXA_2_, MMPs and TF (199, 200). Previous studies indicated that cancer cells with different metastatic potentials had varying abilities to induce TCIPA (201). Zarà et al. explored the molecular pathways of TCIPA formation in breast cancer cells and colorectal cancer cells (202). They made the following discoveries: (1) Plasma was the indispensable environment for the interaction between cancer cells and platelets. (2) Cancer cells interacted with platelets and thus induced thrombin generation, leading to platelet aggregation. (3) Cancer cells regulated TCIPA mainly through binding of fibrinogen to integrin αIIbβ3 on activated platelets. Integrin αIIbβ3 outside-in signaling stimulated phospholipase C (PLC) and Rap1b-GTP and subsequently expedited platelet activation. (4) TCIPA was supported by ADP and its P2Y12 receptor on platelet surface. (5) Different breast and colorectal cancer cell lines triggered platelet aggregation in the same manner, suggesting that the type and metastatic phenotype of cancer cells didn’t make a striking difference in the formation of TCIPA. However, whether other types of cancer adopt the similar ways to induce TCIPA still needs to be studied.

### 4.3 Immune Suppression

CTCs surrounded by activated platelets can escape from innate immune surveillance and cause distant hematogenous metastasis (203). It is known that natural killer (NK) cells play a key role in antitumor immunity (204) while platelets are able to impair NK cell antitumor reactivity in different ways. First of all, platelets could help CTCs evade immune recognition through transferring platelet-derived MHC class I to CTCs (205). Secondly, platelets released TGFβ to downregulate immunoreceptor NKG2D on NK cells (206). Thirdly, ectosomes released from platelets were reported to cause NK cell disfunction by suppressing the expression of NK cell surface receptors (NKG2D, NKp30, DNAM-1) in a TGFβ1-dependent way (207). TGFβ1 in the ectosomes increased miR-183 and thus decreased DNAX activating protein 12 kDa (DAP12), leading to the disturbance of NK functions and downstream signal transduction (208). Fourthly, platelet-derived TGFβ1 induced Foxp3 expression in conventional CD4+ T cells and converted them into regulatory T cells that were capable of killing activated T cells (209). Of note, constitutive expression of TGFβ-docking receptor Glycoprotein A Repetitions Predominant (GARP) in platelets activated TGFβ and augmented the immunosuppressive effects on cancer cells. Thus, platelet inhibition can potentially reinforce adoptive T cell therapy (210). In headneck squamous cell carcinoma, platelets inhibited T cell proliferation, cytokine production (IFN-γ, TNF-α) of CD4+ T Cells and decreased PD-1 expression on CD4+ and CD8+ T cells (211). Platelet-derived PD-L1 disturbed T cell functions and promoted PD-L1 negative tumor growth (212).

A more recent study demonstrated that platelets also facilitated the release of NKG2D ligands MICA and MICB from tumor cells and modulated NKG2D expression on NK cells (213), in a process that platelet-derived ADAM10 (a member of the disintegrin and metalloproteinase family of proteins) may be involved (214). Similarly, platelets could also decrease expression of CD112 and CD155 on tumor cells as well as their associated receptors CD226 and CD96 on NK cells. As a result, tumor cells were protected from NK cell recognition and cytotoxicity (213). Immunomodulatory TNF family members, such as glucocorticoid-induced TNF receptor-related ligand (GITRL) (215), receptor activator of NF-κB ligand (RANKL) and Oxford 40 ligand(OX40L) were upregulated in activated platelets from cancer patients, indicating that they were possibly involved in tumor pathophysiology (216). Platelet-derived GITRL (217) and RANKL (216) both induced NK cell inhibition *via* interacting with their specific receptors on NK cells (GITR, RANK, respectively). Recently, Zhou et al. showed that GITRL overexpression of platelets was substantially associated with tumor-derived soluble factors such as TGFβ (215). These studies prove that platelets boost CTCs’ survival in the process of hematogenous metastasis by suppressing innate and adaptive immunity.

### 4.4 Apoptosis Resistance

CTCs have to overcome detachment-induced apoptosis (namely anoikis) for survival in the circulation. Haemmerle et al. recently elucidated that platelets induced anoikis resistance and metastasis by activating Yes-associated protein 1 (YAP1) *via* the RhoA-MYPT1-PP1 axis (218). Moreover, PDGF could mediate anti-apoptotic properties of fibroblasts *via* the Ras/PI (3)K/Akt/IKK/NF-κB pathway (219). In pancreatic cancer, it was found that platelet-derived growth factor-BB enhanced anoikis resistance and cell migration through YAP signaling (220). Apoptosis signal-regulating kinase 1 (Ask1) is an upstream kinase of the stress-induced mitogen activated protein kinase (MAPKs) pathway. Deficiency of Ask1 impaired platelet granule secretion and TXA2 generation and protected mice from thrombosis (221). Furthermore, the Ask1-JNK/p38 axis also activated ADP receptor P2Y_12_ on platelets to augment tumor metastasis to the lung (222). Intriguingly, binding of platelet TSP1 to the calreticulin/LRP1 complex protected mouse embryo fibroblasts (MEFs) from anoikis *via* the PI3K/Akt signaling pathway (223). Nevertheless, it remains unknown whether platelet-produced TSP1 is involved in anoikis resistance of CTCs.

### 4.5 Platelet–Related Chemoresistance

Increasing evidence indicates that human platelets are associated with chemoresistance of cancer cells. Advanced gastric cancer patients with platelet aggregation have a higher rate of chemoresistance (58.3%) than those without platelet aggregation (20.0%) (224). Primary tumor cells surrounded by platelets exhibited EMT-like morphological changes and resisted some common anticancer drugs (225). It has been established that platelets promote the EMT process of cancer cells, which plays an important role in drug-resistance (226, 227). EMT-related transcription factors such as Snail (228, 229) and Slug (229, 230) are involved in chemotherapy resistance. A recent study has suggested that platelet-derived ADP and ATP increased the expression level of Slug and subsequently modulate human equilibrative nucleoside transporter 1 and cytidine deaminase. As a consequence, they enhanced proliferation and survival of pancreatic ductal adenocarcinoma cells in the presence of gemcitabine (231). In NSCLC, incubation with platelets could prominently relieve the cisplatin-induced inhibition of cancer cell proliferation and angiogenesis. Platelets prevented caspase-3 activation and reduced cancer cell apoptosis through Akt/Bad/Bcl-2 signaling (232). In addition, platelet-derived chemokine RANTES and TSP1 (233) both increased the survival of paclitaxel-treated cancer cells (234). Casagrande et al. suggested that platelet-secreted factors ((EGF, PDGF, TGF-β, IGF and CCL5) protected cancer stem cells from paclitaxel, cisplatin and carboplatin (235). All above data prove the involvement of platelets in cancer chemoresistance. Understanding the mechanisms underlying the platelet–related chemoresistance will help to solve a major problem in anticancer drug therapy.

Radziwon-Balicka et al. tried to explain the possible reasons why platelets increase the survival of colonic and ovarian adenocarcinoma cells in the presence of 5-fluorouracil and paclitaxel (234). According to their experimental data, they surmised that platelets were capable of protecting cancer cells from anticancer drug-induced apoptosis and cell cycle inhibition. Platelets facilitated DNA repair processes and the expression of p38 and JNK-p54 MAPKs (234) that mediated proliferation, differentiation, survival and migration (236).

## 5 Platelets in Cancer Therapy

In view of the close relationship between platelets and cancer development, there are two major strategies for platelet-targeted cancer therapies. One is to develop antiplatelet drugs while the other is to transform the platelets themselves into drug delivery vehicles.

### 5.1 Antiplatelet Agents

Considering the important role of platelets in cancer development and dissemination, antiplatelet agents seem to be a promising adjuvant strategy for cancer treatment. At present, the most studied drug is aspirin, a cyclooxygenase 1 (COX) inhibitor. As a nonsteroidal anti-inflammatory drug (NSAID), aspirin can inhibit COX-1 in platelets to reduce PGE_2_ and TXA_2_, subsequently attenuating the tumor metastasis (237). Long-term clinical trials showed that taking aspirin daily (>75mg) for years brought down incidence and mortality of colorectal cancer, especially the proximal colon cancer (238). Aside from colorectal cancer, aspirin use is capable of decreasing the risk of gastric cancer (239), pancreatic cancer (240) and cholangiocarcinoma (241) and increasing the survival of advanced-stage prostate cancer (242), breast cancer (243) and endometrial cancer (244). However, some other clinical studies showed that aspirin had no effects on cancer risk (245) or cancer-specific death (246, 247). Therefore, more randomized clinical trials are needed to validate the preventive and therapeutic effects of aspirin on cancer treatment.

Other antiplatelet drugs including antagonists of ADP receptor P2Y_12_, integrins (αIIbβ3, α2β1), P-selectin, CLEC-2 (7) are all being investigated. Of note, there are some natural materials acting as antiplatelet agents. Irfan et al. recently identified *Eisenia bicyclis* as a potential anti-thrombotic agent for cardiovascular disease (CVD) and possibly cancer with fewer side effects. *Eisenia bicyclis* inhibited ADP-induced platelet aggregation by suppressing PI3K/Akt signaling and MAPK activation in a dose-dependent manner (248). Norcantharidin (NCTD), a demethylated analogue of cantharidin, is clinically utilized for cancer chemotherapy in China for years (249). It was found to have powerful antiplatelet effects through suppression of integrin aIIbβ3 mediated outside-in signaling in human platelets (250).

However, most of these antiplatelet agents are still in the early stages and lack statistical power for wide clinical application. Moreover, use of antiplatelet agents likely leads to thrombocytopenia and bleeding complication (8, 251). Some studies even suggested that long-term inhibition of platelet function could pose a hazard in return (252, 253). In fact, it was found that 4T1 metastatic breast cancer-bearing mice had reduced survival when treated with dual platelet inhibitors clopidogrel and aspirin (254).

Fortunately, some novel platelet inhibitors have appeared with a more favorable safety profile. Investigators have found that ruthenium complexes with antiplatelet properties (255) exerted higher efficacy and lower side effects in cancer therapy (256, 257), in comparison with standard cisplatin and carboplatin therapies (258, 259). Thanasekaran et al. have reviewed the molecular mechanisms of ruthenium compounds in repressing platelet activation (255). Ruthenium complexes TQ3 (260), TQ5 (261) and TQ6 (262) could reduce granule secretion and hinder platelet activation and aggregation. More importantly, ruthenium compounds exhibited higher cytotoxicity in cancer cells than normal cells (263) and showed improved safety without increased LDH activity in platelets (260–262). As is known, human platelet-expressed NADPH oxidases (NOX) accelerate reactive oxygen species (ROS) generation and activate platelets (264). Recently, NOX2 inhibitor Phox-I was observed to disrupt platelet activation without altering the hemostatic response to injury. It was capable of reducing platelet ATP secretion, calcium level and restricting PI3K signaling and p38-MAPK in thrombin-stimulated platelets (265).

The above data demonstrate that antiplatelet drugs have great potentialities for clinical application. In consideration of physiological functions of platelets, how to reduce the side effects of antiplatelet agents will be a pivotal issue in the future.

### 5.2 Platelet-Based Drug Delivery System

Recently, Lu et al. provided a comprehensive description of platelet-mediated drug delivery systems that include platelet hitchhiking, membrane coating, platelet engineering, synthetic platelet fabrication and platelet-triggered drug release (266). By binding to platelets through targeting platelet adhesion molecules (e.g. GPIIb/IIIa, P-selectin, phosphatidylserine), nanoparticles with anti-thrombosis drugs (267) or anticancer drugs (268) could become powerful targeted drugs (266). However, Chen et al. revealed that the platelet targeting effects of magnetic nanoparticles (MNPs) varied due to distinct tumoral microenvironment. MNPs were effective in breast cancer with adequate blood supply and low extracellular matrix (ECM) expression, but not in ischemic pancreatic cancer (268).

Synthetic silica particles with platelet membrane (PM) can also be used to deliver anti-cancer drugs to CTCs. Tumor necrosis factor–related apoptosis inducing ligand (TRAIL) on the particle surface was shown to specifically induce apoptosis of cancer cells (269). Hu et al. designed a PM-coated core-shell nano-vehicle with TRAIL and Dox (TRAIL-Dox-PM-NV). Through the interaction between P-selectin on PM and CD44 receptors on the cancer cells, TRAIL-Dox-PM-NV aggregated at the surface of CTCs and suppressed their survival and spreading (270). Similarly, a newly PM-decorated nanoparticle that incorporated both DOX and a photothermal agent, indocyanine green (ICG), had the capability to track CTCs in lymph nodes and blood through P-selectin-CD44 interplay and eliminate CTCs by releasing DOX and ICG. It exhibited strong inhibitory effects on orthotopic tumor growth and metastasis in breast cancer (271).

In platelet engineering, platelets could load drugs *via* platelet surface modification, platelet phagocytosis, or genetic manipulation of megakaryocytes (266). DOX loaded-platelets could augment therapeutic effects of lymphoma with less cardiotoxicity (272), while interferon-γ induced protein 10 (IP10)-loaded platelets could inhibit tumor growth and increase anti-tumor immunity by reducing regulatory T cells in melanoma model (273). Zhang et al. constructed engineered platelets expressing the programmed cell death protein 1 (PD-1) to prevent tumor relapse after surgical resection. These recombinant platelets could aggregate at the surgical wound sites and eradicate residual tumor cells by reverting CD8+ T cells. Moreover, cyclophosphamide carried by such platelets could exhaust regulatory T cells and promote the anticancer effects of CD8+ T cells (274). Intriguingly, investigators found that the conjugate of hematopoietic stem cells (HSCs) and platelets decorated with anti-PD-1 antibodies (aPD-1) could enter the bone marrow due to the homing capability of HSCs. Then platelets were activated in leukemia microenvironment and released aPD-1 to enhance immune response in mice with acute myeloid leukaemia (275). They further genetically modified mouse MK progenitor cells that produced PD-1-presenting platelets under stimulation. These platelets could effectively gather at tumor resection site *via* thrombosis and PD-1/PD-L1 interaction and thus inhibit the tumor recurrence. The therapeutic potency was enhanced when PD-1-presenting platelets were loaded with cyclophosphamide (276). In addition, Li et al. developed a strategy that combined Vadimezan and aPDL1-loaded platelets to inhibit tumor metastases. Vadimezan disrupted tumor blood vessels and then recruited aPDL1-conjugated platelets at the tumor site, leading to immune activation and enhanced antitumor effects (277).

Recently, Papa et al. developed detergent-extracted human modified platelets (platelet decoys) that retained binding capacity but couldn’t aggregate in response to platelet agonists. Importantly, decoys inhibited aggregation and adhesion of natural platelets and then halted blood clot formation and cancer spread, which could be reversed immediately by transfusing functional platelets. Papa’s team believed that decoys had the potential to be loaded with drugs and specifically target thrombosis, tumors or CTCs (278).

These studies support the notion that modified platelets or particles that mimic platelets are able to deliver antineoplastic drugs or antitumor proteins to CTCs with prolonged circulation time, achieving potent antitumor effects. Since platelets also play an important part in thrombosis formation, this method may also be useful for cardiovascular disease. Nonetheless, since platelets can be attracted to damaged vasculature and carry out their physiological functions, platelet-based drug delivery system may cause off-target effects. In this regards, it should be noted that there are reports demonstrating that platelets were innate immune cells and exhibited some anti-cancer properties (279), protected endothelial barrier and decreased cancer cell intravasation and extravasation (280). Above all, considering the close interaction between platelets and cancer, platelets can be converted to anti-cancer drug delivery vehicles. Compared with other tumor-targeting nanoparticles, platelet-derived vehicles have some remarkable advantages including prolonged circulation time and large cargo capacity (281). Large clinical trials are warranted to confirm the therapeutic values of platelets and platelet-derived particles.

## 6 Concluding Remarks

**Figure 1:**
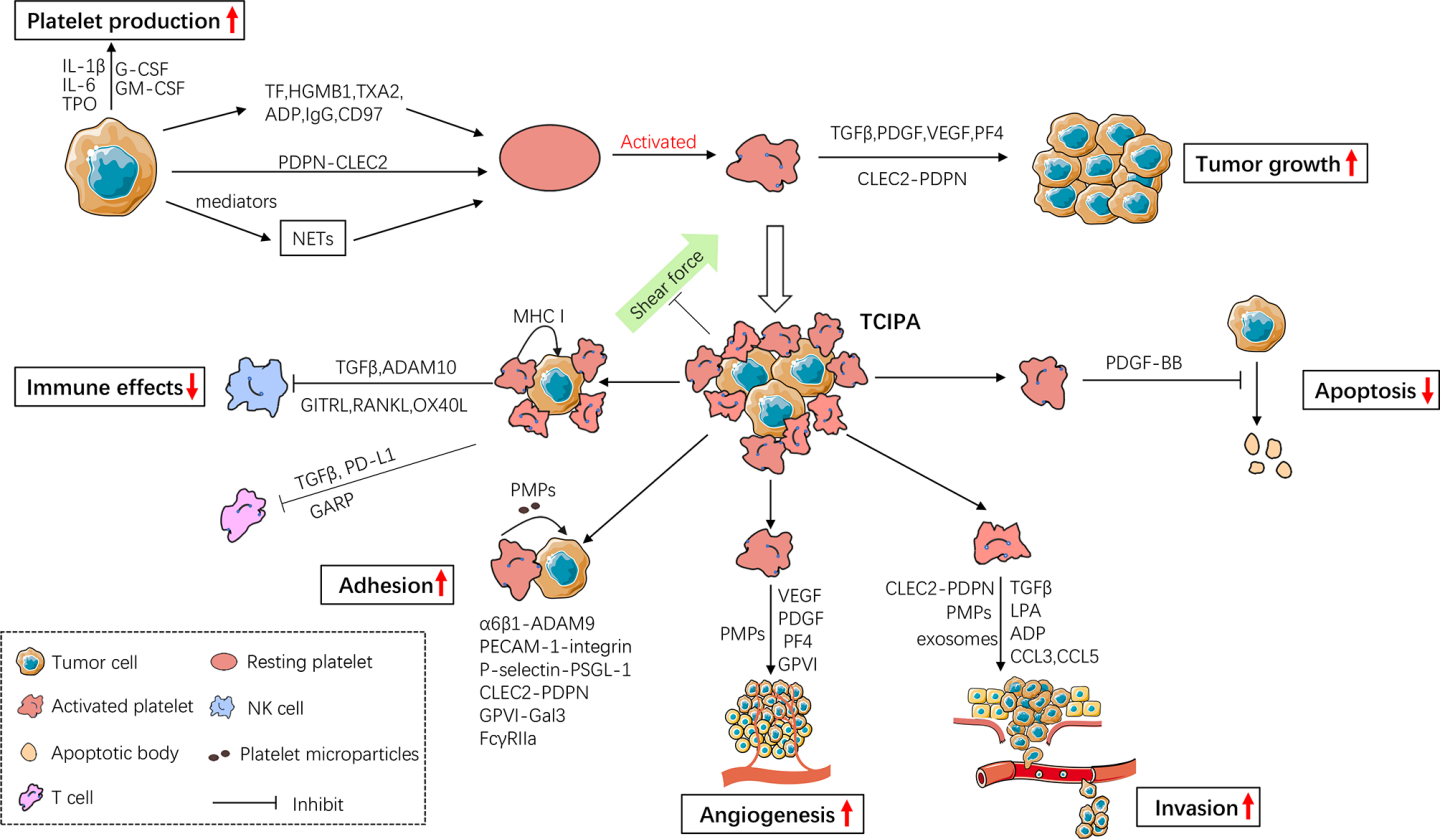
The bidirectional interaction between cancer cells and platelets. Cancer cells promote platelet production and activation, while activated platelets release a number of mediators to facilitate tumor growth and cancer cell metastasis. Activated platelets prevent circulating cancer cells (CTCs) from shear flow, immune surveillance and apoptosis, thus enhancing CTC survival in circulation. They also facilitate CTC adhesion, angiogenesis and invasion thereby enhancing metastasis. IL-1β, Interleukin-1β; IL-6, Interleukin-6; TPO, thrombopoietin; G-CSF, Granulocyte- Colony-Stimulating Factor; GM-CSF, Granulocyte-Macrophage-Colony-Stimulating Factor; TF, Tissue Factor; HGMB1, High-Mobility Group Box1; TXA2, Thromboxane A2; IgG, immunoglobulin G; PDPN, Podoplanin; CLEC2, C-type lectin receptor type 2; NETs, Neutrophil Extracellular Traps; TGFβ, Transforming Growth Factor β; PDGF, Platelet-Derived Growth Factor; VEGF, Vascular Endothelial Growth Factor; PF4, Platelet Factor 4; TCIPA, Tumor Cell-Induced Platelet Aggregation; MHC I, MHC class I; ADAM10, Disintegrin And Metalloproteinase Domain-Containing Protein 10; GITRL, Glucocorticoid-Induced TNF Receptor-Related Ligand; RANKL, Receptor Activator of NF-κB Ligand; OX40L, Oxford 40 Ligand; GARP, Glycoprotein A Repetitions Predominant; PD-L1, Programmed Cell Death-Ligand 1; PMPs, Platelet Microparticles; PECAM-1, Platelet-Endothelial Cell Adhesion Molecule-1; PSGL-1, P-selectin Glycoprotein Ligand-1.

Overwhelming evidence supports an auxiliary positive role of platelets in promoting both primary cancer and metastatic cancer. As illustrated in **Figure 1**, the interaction between tumor cells and platelets is bidirectional. On one hand, as a component of the tumor microenvironment, platelets are educated by cancer cells to facilitate survival and dissemination of cancer cells. On the other hand, cancer cells induce platelet production, activation and aggregation to increase the risk of thrombosis in cancer patients. Studying the interplay of platelets with cancer cells has major implications for diagnosis and treatment of cancers. Platelet counts, RNA profile, proteome and platelet-derived factors/microparticles in cancer patients can be used for early cancer detection, prognosis monitoring and assessment of chemotherapy curative effects. Antiplatelet and anti-thrombosis drugs have promising prospects for cancer treatment. Moreover, combination of platelet inhibition and other therapy strategies (such as photothermal therapy) may achieve synergistic and potent anticancer effects (282). However, many questions still remain to be answered, such as how to control the degree of platelet inhibition without disrupting their physiological functions and what kinds of patients are suitable for using antiplatelet agents. Platelet-based drug delivery system is an innovative method for cancer therapy, but how to avoid off-target effects is the greatest challenge.

## Author Contributions 

All authors listed were contributed to the manuscript writing and revising. They all approved the submitted version.

## Funding

The work was supported by Guangdong Provincial Key Laboratory of Digestive Cancer Research (No. 2021B1212040006), National Natural Science Foundation of China (NSFC, No. 82000150), Shenzhen Science and Technology Innovation Commission (JCYJ20190809172403604, JCYJ20190809164617205) and Sanming Project of Medicine in Shenzhen (No. SZSM202011011).

## Conflict of Interest

The authors declare that the research was conducted in the absence of any commercial or financial relationships that could be construed as a potential conflict of interest.

## Publisher’s Note

All claims expressed in this article are solely those of the authors and do not necessarily represent those of their affiliated organizations, or those of the publisher, the editors and the reviewers. Any product that may be evaluated in this article, or claim that may be made by its manufacturer, is not guaranteed or endorsed by the publisher.
